# The Application of the Essential Oils of *Thymus vulgaris* L. and *Crithmum maritimum* L. as Biocidal on Two *Tholu Bommalu* Indian Leather Puppets

**DOI:** 10.3390/plants10081508

**Published:** 2021-07-22

**Authors:** Giulia D’Agostino, Belinda Giambra, Franco Palla, Maurizio Bruno, Natale Badalamenti

**Affiliations:** Department of Biological, Chemical and Pharmaceutical Sciences and Technologies (STEBICEF), Università degli Studi di Palermo, Viale delle Scienze, Ed. 17, I-90128 Palermo, Italy; giuliadagostino@outlook.com (G.D.); info@belindagiambra.it (B.G.); franco.palla@unipa.it (F.P.); natale.badalamenti@unipa.it (N.B.)

**Keywords:** *Thymus vulgaris*, *Crithmum maritimum*, leather artifacts, essential oils, anti-bacterial activity

## Abstract

The chemical profile of the *Thymus vulgaris* (Lamiaceae) essential oil (EO) was investigated in order to evaluate its biological properties against microorganisms affecting two *Tholu Bommalu*, typical Indian leather puppets stored at the International Puppets Museum “Antonio Pasqualino” of Palermo, Italy. A GC–MS analysis, using both polar and apolar columns, was used to determine the chemical composition of the essential oil. The aim of this study was to evaluate the antimicrobial effectiveness of the *Thymus vulgaris* and *Crithmum maritimum* essential oils in vapor phase to disinfect heritage leather puppets. Pieces of leather artifacts that were affected by different bacterial colonies were exposed to EO under vacuum and static evaporation conditions. The results presented showed that the vaporization of essential oil was an efficient method in the disinfection of natural skins, eradicating microorganism in short times. *T. vulgaris* EO in the 50% solution showed excellent inhibitory activity against isolated bacteria with both methods, but the obtained results suggest that the vacuum method allowed for faster exposition of the artifacts to the biocide. Furthermore, the biocidal properties of the essential oil of a Sicilian accession of *Crithmum maritimum* (Apiaceae) aerial parts were compared and investigated. The results of the latter essential oil showed a poor activity against the isolated micro-organisms.

## 1. Introduction

Biodeterioration of cultural heritage causes different alteration processes affecting the constitutive materials of artworks. In particular, fungi and bacteria are able to colonize different artworks comprised of natural materials through aerosol pollution, representing complex problems for conservation by causing a loss of mechanic resistance and deterioration of pictorial layers. Moreover, inadequate exposure to specific thermo-hygrometric parameters can increase the concentrations of microbial colonies, enhancing the deterioration process. For the purpose of inhibiting the biological colonization, different chemical biocides, such as permethrin or/and benzalkonium chloride (BAK), are frequently used. These products are usually toxic for humans and the environment. Hence, in the last decade, essential oils have been applied in order to combat cultural heritage biodeterioration as an eco-friendly solution [1,2,3,4,5,6,7,8,9,10,11,12]. To prevent biodeterioration caused by fungi and bacteria, objects must first be disinfected. The requirements for a disinfectant include the ability to inhibit the growth and metabolic activity of microorganisms without adversely affecting the material. Currently, fumigation with ethylene oxide is the most popular method for disinfecting fabrics, papers and leathers. However, this gas is an irritant and a dangerous human carcinogen, and its use should be avoided [13].

However, the disinfection process by using volatile compounds, such essential oils (EOs), can require a long time, so the purpose of this work was to find a way to accelerate this. During the evaporation process of a specific liquid, the number of molecules escaping from the liquid increases every second and those that condense back into the liquid also increase. Reaching thermodynamic equilibrium, the vapor phase exerts a pressure on its condensed phase, which is called “vapor pressure” [14]. The speed with which the evaporation occurs is proportional to the vapor pressure of the liquid. The purpose of a vacuum pump applied to a closed system is to maintain a lower pressure than that of the atmosphere. In order to make our disinfection process faster, a vacuum pump was connected to a closed system, ensuring a higher evaporation of the EO contained inside.

To investigate a suitable alternative to traditional synthetic biocides, we decided to analyze the biocidal properties of two different essential oils: *Thymus vulgaris* L. (Lamiaceae) and *Crithmum maritimum* L. (Apiaceae).

*Thymus* EO was selected due to its microbial properties, which have previously been reported in several studies [15,16,17,18,19,20,21]. In particular, the *Thymus vulgaris* essential oil has been shown to be quite effective against several microorganisms [22,23].

In addition, the essential oil of *Crithmum maritimum* has been largely investigated [24]. Its antimicrobial properties have been studied [25,26], showing its antioxidant, anti-inflammatory, vermifuge and antifungal potentials. In particular, the essential oil’s capacity to inhibit two important virulence factors in *Candida albicans*, *Cryptococcus neoformans* and several dermatophytes and *Aspergillus* spp has been demonstrated.

There are no reports on the use of *Thymus* essential oil for leather disinfection to date. Thus, the main purpose of this study was to undertake the first study of the application of the *Thymus* essential oil to heritage leather disinfection under a vacuum system. Consequently, in the context of our ongoing research on endemic Sicilian plants [27,28,29] and the biological activity of essential oils [30,31,32], and in consideration of the important antibacterial properties of the essential oil *Thymus* demonstrated in the aforementioned articles, we decided to utilize the EO of *Thymus vulgaris* as natural biocide against bacteria affecting two *Tholu Bommalu*, typical Indian leather puppets, which were stored at the International Puppets Museum “Antonio Pasqualino”. We exploited the high vacuum inside ad hoc chambers in order to speed up the disinfection process. The activity of the essential oil of *T. vulgaris* was then compared with that of Sicilian *Crithmum maritimum* EO. These results were compared with the results of using a conventional synthetic biocide, benzalkonium chloride, as a disinfectant.

## 2. Results and Discussion

### 2.1. Gas Chromatography and Mass Spectrometry (GC-MS) Analysis of the Essential Oil

The chemical composition of the essential oil of *Thymus vulgaris* was analyzed by GC-MS analysis and is reported in Table 1. Sixteen compounds, divided into three classes, were identified, accounting for 97.97% of the total composition. In terms of compound classes, monoterpene hydrocarbons (49.96%) dominate the EO, with *p*-cymene as the most abundant compound (35.96%), followed by terpinen-4-ol (10.29%) and *α*-terpinene (8.85%). Oxygenated monoterpenes are also dominant (43.67%) with thymol (25.38%). In contrast, sesquiterpene hydrocarbons accounted for only 4.34%; no oxygenated sesquiterpenes were identified in the chromatogram of the EO. Comparing different samples of *T. vulgaris* from Saudi Arabia [22] and France and Serbia [33] to our results, we found them to also be rich in not only thymol, *p*-cymene and *α*-terpineol but also in camphene and caryophyllene.

**Table 1:** Chemical composition of *T. vulgaris* essential oil.

No.	Compounds	LRI ^a^	LRI ^b^	Area (%)	Ident. ^c^	Ref.
1	*β*-Pinene	974	1070	0.08	1, 2, 3	
2	*cis*-Carane	978	1033	0.90	1, 2	
3	*β*-Myrcene	990	1079	2.54	1, 2, 3	
4	3-Carene	1011	1129	0.48	1, 2	
5	*α*-Terpinene	1018	1140	8.85	1, 2, 3	
6	*p*-Cymene	1021	1224	35.96	1, 2, 3	
7	(3*E*)-3-Ethyl-2,5-dimethyl-1,3-hexadiene ^d^	1031	976	0.04	1, 2	
8	Eucalyptol	1035	1211	1.56	1, 2, 3	
9	Terpinolene	1084	1264	1.11	1, 2	
10	*β*-Linalool	1098	1498	5.99	1, 2, 3	
11	*trans*-Pinocarveol	1139	1666	0.45	1, 2	
12	Terpinen-4-ol	1177	1612	10.29	1, 2	
13	Thymol	1267	2156	25.38	1, 2, 3	
14	*α*-Copaene	1376	1487	0.14	1, 2	[34]
15	Caryophyllene	1419	1598	4.13	1, 2, 3	
16	*δ*-Cadinene	1530	1722	0.07	1, 2	
	Monoterpene Hydrocarbons	49.96				
	Oxygenated Monoterpenes	43.67				
	Sesquiterpene Hydrocarbons	4.34				
	Total	97.97				

^a^: retention index on a HP-5MS apolar column; ^b^: retention index on a DB-Wax polar column. ^c^: 1 = retention index identical to bibliography; 2 = identification based on comparison of MS; 3 = retention time identical to authentic compounds; ^d^: tentative identification. Compounds are classified in order of linear retention time of apolar column.

The chemical composition of the essential oil obtained from *Crithmum maritimum* was analyzed by GC-MS analysis and is reported in Table 2. Forty compounds, divided into four classes, were identified, accounting for 90.03% of the total oil. In terms of compound classes, monoterpene hydrocarbons (45.08%) dominate the EO, with *β*-myrcene as the most abundant compound (13.66%), followed by *p*-cymene (11.67%) and *β*-phellandrene (6.57%). Oxygenated monoterpenes are also dominant (40.03%) with thymol acetate (14.38%). In contrast, hydrocarbon and oxygenated sesquiterpenes accounted for only 1.94% and 0.50%, respectively.

**Table 2:** Chemical composition of *C. maritimum* essential oil.

No.	Compounds	LRI ^a^	LRI ^b^	Area (%)	Ident. ^c^	Ref.
1	*α*-Pinene	932	1019	5.51	1, 2, 3	
2	Camphene	944	1060	5.16	1, 2, 3	
3	Sabinene	966	1114	0.21	1, 2	
4	*β*-Pinene	974	1070	0.55	1, 2, 3	
5	*β*-Myrcene	990	1079	13.66	1, 2, 3	
6	*α*-Phellandrene	998	1166	0.88	1, 2	
7	*p*-Cymene	1021	1224	11.67	1, 2, 3	
8	*β*-Phellandrene	1022	1201	6.57	1, 2	
9	*γ*-Terpinene	1049	1233	0.87	1, 2,	
10	*β*-Linalool	1098	1498	0.43	1, 2, 3	
11	*cis*-2-Menthenol	1118	1527	0.39	1, 2	[35]
12	Camphor	1122	1501	0.27	1, 2, 3	[36]
13	3-Methylbutyl pentanoate	1134	1345	0.16	1, 2	
14	*trans*-2-Menthenol	1136	1561	0.56	1, 2	[37]
15	Camphene hydrate	1148	1583	0.09	1, 2	
16	Terpinen-4-ol	1177	1612	3.52	1, 2	
17	Pentyl pentanoate^d^	1185	1402	0.51	1, 2	
18	*trans*-3(10)-Caren-2-ol	1194	1525	0.18	1, 2	[38]
19	1-Methylhexyl butanoate	1197	1371	0.63	1, 2	[39]
20	Carvone	1218	1709	0.24	1, 2, 3	
21	*cis*-Carveol	1222	1820	0.19	1, 2	
22	Thymol methyl ether	1235	1587	1.23	1, 2	
23	2-Methylcyclohexyl butyrate ^d^	1241	1407	0.37	1, 2	
24	4,7-Dimethylbenzofuran	1260	1619	0.54	1, 2	[40]
25	Thymol	1267	2156	2.62	1, 2, 3	
26	Bornyl acetate	1270	1555	2.69	1, 2	
27	Piperitone	1277	1730	0.40	1, 2	
28	2-Undecanone	1295	1614	0.27	1, 2	
29	Thymol acetate	1352	1813	14.38	1, 2, 3	
30	4-Cyclohexyl-2-butanone ^d^	1371	1823	0.10	1, 2	
31	Damascenone	1385	1810	0.48	1, 2	
32	*α*-Ionol	1395	1881	0.29	1, 2	
33	1,4-Dimethoxy-2-tert-butylbenzene	1408	1871	1.23	1, 2	[41]
34	*α*-Methylbenzyl propionate ^d^	1412	1853	0.99	1, 2	
35	2-Methyl-6-(2-propenyl)phenol ^d^	1423	1889	3.30	1, 2	
36	Geranyl acetone	1456	1859	0.30	1, 2	
37	Bornyl butyrate	1463	1760	1.30	1, 2	
38	*γ*-Cadinene	1521	1776	1.94	1, 2	
39	2,3,4-Trimethylacetophenone	1548	1882	5.35	1, 2	
	Monoterpene Hydrocarbons	45.08				
	Oxygenated Monoterpenes	40.03				
	Sesquiterpene Hydrocarbons	1.94				
	Oxygenated Sesquiterpenes	0.50				
	Others	2.48				
	Total	90.03				

^a^: retention index on a HP-5MS apolar column; ^b^: retention index on a DB-Wax polar column. ^c^: 1 = retention index identical to bibliography; 2 = identification based on comparison of MS; 3 = retention time identical to authentic compounds; ^d^: tentative identification. Compounds are classified in order of linear retention time of apolar column.

### 2.2. Antimicrobial Activity

In order to test the antimicrobial activity of *T. vulgaris* and *C. maritimum* EOs against bacteria species isolated from Tholu Bommalu, an agar disk diffusion method (ADD) was performed (results are listed in Table 3). As far as the *T. vulgaris* EO was concerned, *Georgenia* sp. isolated colonies were the most susceptible to the oil; in this case, the antimicrobial activity was so high that the inhibition halos were confluent. Every bacterial colony showed a relevant sensitivity to both EO solutions (50% and 100%), which were able to produce inhibition halos of up to 33 mm. In contrast, the *C. maritimum* essential oil did not show inhibition halos, except in the case of *Bacillus* sp., although these inhibition halos were smaller than those of *T. vulgaris*.

**Figure 1:**
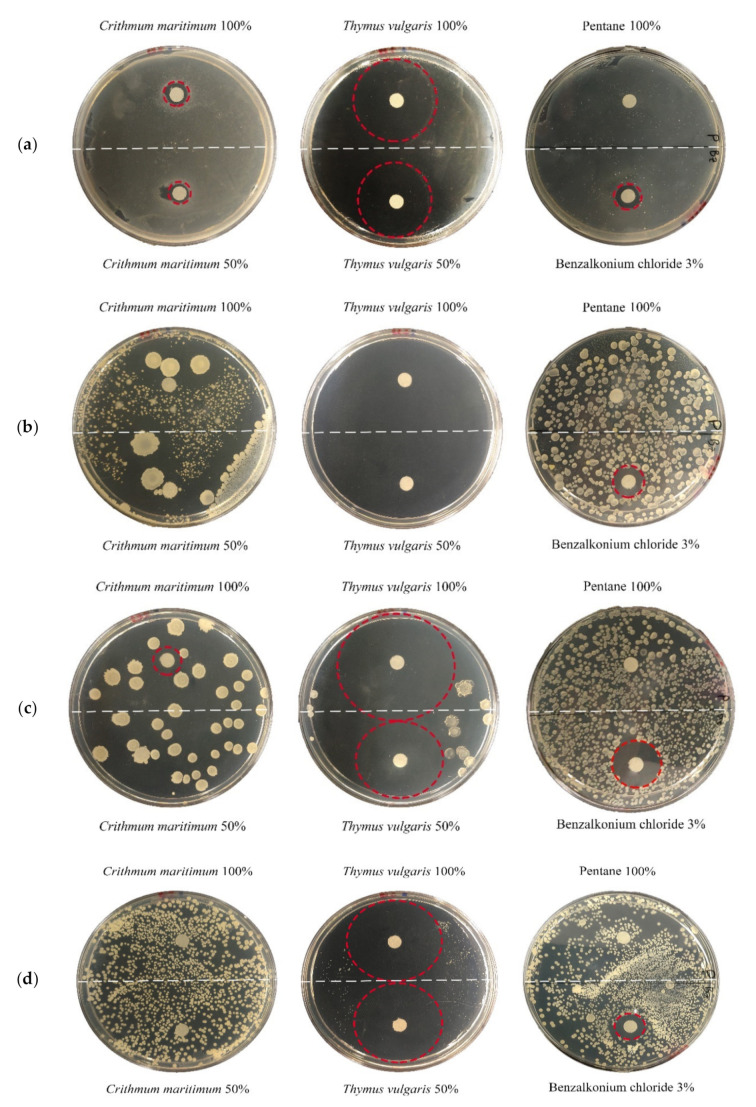
Inhibition halos highlighted using ADD method with *T. vulgaris* and *C. maritimum* EOs and control BAK against (**a**) *Bacillus* sp., (**b**) *Georgenia* sp., (**c**) *Ornithinibacillus* sp. and (**d**) *Streptomyces* sp.

The antimicrobial activity of the *T. vulgaris* EO was displayed against all isolated colonies and clearly had a greater effect than that of the controls (benzalkonium chloride (BAK), the reference biocide, and pentane, as shown in Figure 1). Completely in contrast was the antimicrobial activity of the *C. maritimum* EO, which produced inhibition halos smaller than those produced by the controls.

The biocide properties of the *T. vulgaris* EO are in agreement with its chemical composition. Several studies highlighted how EOs rich in phenolic compounds, such as thymol or carvacrol, have a strong antimicrobial activity. In particular, thymol seems to be bioactive against more than twelve different bacterial colonies [21,42].

### 2.3. Use of a Vaccum Chamber for the Disinfection Process

Considering the huge presence of microbial colonies on *Tholu Bommalu* and the fragility of the parchment support, the aim was to find a disinfection process that was minimally invasive but also fast, allowing replicability in the future for the entire collection of Indian shadow puppets stored in the International Puppets Museum, “Antonio Pasqualino”.

**Figure 2:**
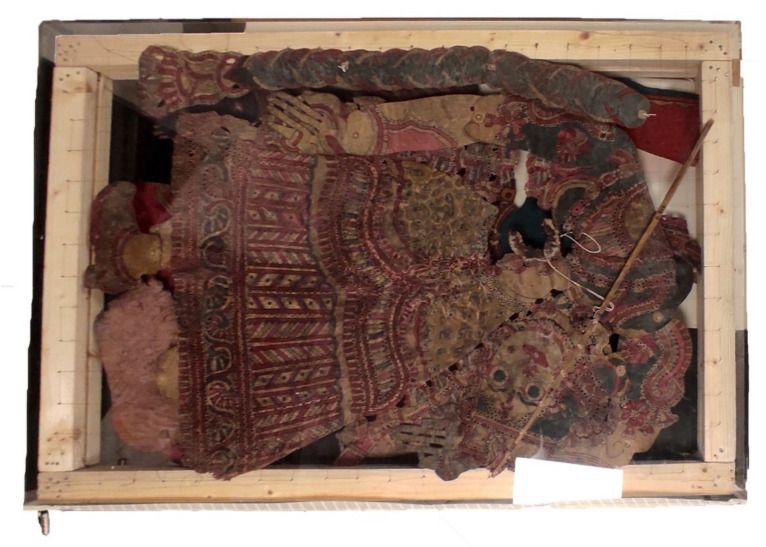
*Tholu Bommalu* exposed to volatile compounds of EO inside the Plexiglas chamber under vacuum.

The study focused on observing the growth of isolated microbial colonies in response to their exposure to volatile compounds of the *Thymus vulgaris* EO, under both normal evaporation conditions and under vacuum inside a Plexiglas chamber (Figure 2).

In order to track the microbial growth on *Tholu Bommalu* fragments affected by bacterial colonies, surface sampling was performed weekly both on the samples in the clean chamber and in the vacuum chamber. The exposure of the fragments to EO volatile compounds lasted a total of five weeks. *T. vulgaris* essential oil manifested a marked reduction in microbial growth over time. This was observed in both the group of samples. However, a clear difference was observed between the two methods, demonstrating that the exposure under vacuum allowed for faster inhibition of microbial growth.

Microbial in vitro cultures were performed on both nutrient agar (Oxoid, Thermo Fisher: 1.0 g Lab-Lemco powder; 5.0 g peptone; 5.0 g NaCl; 15.0 g agar/liter of dH_2_0) and Sabouraud dextrose agar (Oxoid, Thermo Fisher: 40.0 g dextrose; 10.0 g peptone; 15.0 g agar/liter of dH_2_O). Nutrient agar supports a wide range of microorganisms, whereas Sabouraud is mainly used to cultivate fungi or filamentous bacteria.

**Figure 3:**
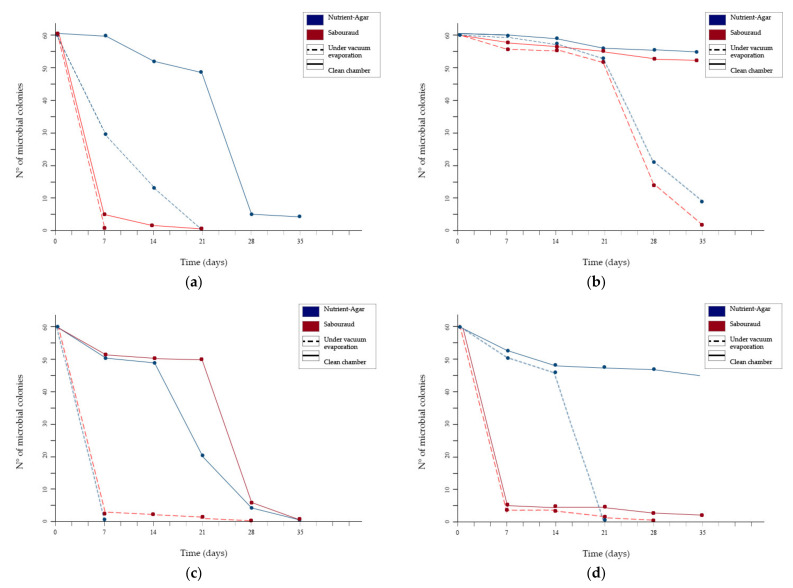
Microbial growth trends over time for fragments “A” (**a**); “B” (**b**); “C” (**c**) and “D” (**d**).

The results from these different culture media were combined and evaluated, revealing the microbial growth trend over time for each fragment (as shown in Figure 3).

In particular, one of the clearest results was related to the colonies sampled from fragments “A” and “C” and grown on nutrient agar, which were significantly reduced in fragment “A” and completely absent in fragment “C” from the first week of exposure to EO volatile compounds. On the contrary, the same colonies exposed in the clean chamber without the vacuum showed confluent growths.

Colonies collected on surfaces of the “A” and “C” fragments showed differences in terms of the microbial load. Specifically, after only one week of exposure to EO volatile compounds under vacuum no microbial growth was observed, whereas microbial growth was still present under ambient conditions, although considerably reduced.

By the third week, the exposure to *T. vulgaris* EO volatile compounds under vacuum conditions resulted in the microbial colonies on “A” and “C” fragments being completely eradicated. In contrast, under environmental conditions, the microbial colonies were still present, revealing a considerable reduction in the microbial load only after the fourth week of the disinfection process.

In regard to fragments “B” and “D”, the microbial colonies present on their surfaces immediately proved to be more resistant to the action of the EO, showing confluent growth in all cases. The first result of microbial growth inhibition was observed from the fourth week of exposure. Under vacuum conditions, the colonies present in “B” were significantly reduced on both nutrient and Sabouraud agar when compared to the same fragments exposed under environmental conditions. Regarding fragment “D”, the microbial growth tested on Sabouraud agar was almost completely eradicated under vacuum conditions and was significantly reduced under environmental conditions. Microbial growth remained confluent on nutrient agar.

Reaching the fifth week of exposure to EO volatile compounds, microbial growth had almost completely stopped under vacuum conditions. The only exception was fragment “B”, although in this case, the microbial load was still reduced. This result was completely in contrast with the exposure under environmental conditions, in which confluent growth was still present after 35 days of disinfection, although only for some taxa.

An important aspect, which must be emphasized, is that a non-topical and direct application on the leather fragments and on the entire artifact using only the vapor pressure of the EO did not cause decolorization processes. This aspect was checked with the aid of a colorimeter (NH300 Colorimeter, 3NH Shanghai Co., Ltd.), evaluating parameters such as total color differences (ΔE), a * (red-green), L * (lightness) and b * (yellow-blue) before and after the EO treatment.

Furthermore, to avoid discomfort and residual odor of the same on the artifact, the latter was subjected to three high vacuum cycles, maintaining stable thermo-hygrometric parameters, in order to eliminate the possible presence of some volatile components of the essential oil.

## 3. Materials and Methods

### 3.1. Essential Oils

The essential oil of *Thymus vulgaris* (100% pure, 100 mL) was purchased from Authentic Oil Co, Unit 1, Little Castle Farm, Raglan, Monmouthshire, NP15 2BX, UK. In this case, the aerial parts of *T. vulgaris* come from Daran, Karaman, Turkey.

The aerial parts of *Crithmum maritimum* were collected in Addaura (Mondello), Palermo, Sicily, Italy (38°11′31″N, 13°20′41″E, 2 m m.s.l.), in June 2020 and a voucher specimen was deposited in STEBICEF Department, University of Palermo (PAL 348/20).

A quantity of 300 g of the aerial parts of *C. maritimum* were subjected to hydrodistillation for 3 h using Clevenger’s apparatus [43]. The oil (yield 2.08% (*v*/*w*)) was dried with anhydrous sodium sulfate, filtered and stored in a freezer at −20 °C until the time of analyses.

### 3.2. GC-MS Analysis of Essential Oil

Analyses of essential oils were performed according to the procedure reported by Rigano et al. [44]. EO analysis was performed using an Agilent 7000 C GC (Agilent Technologies, Inc., Santa Clara, CA, USA) system, fitted with a fused silica Agilent DB-Wax capillary column (30 m × 0.25 mm i.d.; 0.25 μm film thickness) and coupled to an Agilent triple quadrupole Mass Selective Detector MSD 5973 (Agilent Technologies, Inc., Santa Clara, CA, USA). The settings were as follows: ionization voltage, 70 eV; electron multiplier energy, 2000 V; transfer line temperature, 295 °C; solvent delay, 4 min. The other GC analysis was performed with a Shimadzu QP 2010 plus equipped with an AOC-20i autoinjector (Shimadzu, Kyoto, Japan), gas chromatograph equipped with a flame ionization detector (FID), a capillary column (HP-5MS) (30 m × 0.25 mm i.d.; film thickness, 0.25 μm) and a data processor. The oven program was as follows: temperature increase at 40 °C for 5 min at a rate of 2 °C/min up to 260 °C and then isothermal amplification for 20 min. Helium was used as the carrier gas (1 mL min^−1^). The injector and detector temperatures were set at 250 °C and 290 °C, respectively. An amount of 1 μL of each oil solution (3% EO/hexane *v*/*v*) was injected with a split mode. Linear retention indices (LRI) were determined by using retention times of *n*-alkanes (C_8_-C_40_), and the peaks were identified by comparison with mass spectra and by comparison to their relative retention indices with WILEY275 (Wiley), NIST 17 (NIST, The National Institute of Standards and Technology, Gaithersburg, MD, USA), ADAMS (Allured Business Media, Carol Stream, IL, USA) and FFNSC2 (Shimadzu, Kyoto, Japan) libraries.

### 3.3. ADD Control Solutions

Benzalkonium chloride (3% *v*/*v*) (Sigma Aldrich, St. Louis, MO 68178 United States), the reference biocide, and pentane (Sigma Aldrich, 100%, St. Louis, MO 68178 United States), the solvent accounting for fifty percentage of the EO solution, were controls used in agar disc diffusion (ADD) assays [45,46]. ADD assays, performed on nutrient or Sabouraud agar media, were performed twice.

### 3.4. Microbial Taxa

Microbial patina on the leather substrata of *Tholu Bommalu* was sampled by sterile stubs. Distinctive colonies, in morphology and/or pigmentation, were isolated on the nutrient agar in vitro culture. Taxonomical identification was performed by molecular analysis of the 16S rDNA gene or ITS1-2 containing DNA region [47]. Specifically, *Bacillus* sp., *Georgenia* sp., *Ornithinibacillus* sp. and *Streptomyces* sp. were identified as the prevalent genera (Gram + bacteria).

### 3.5. Antibacterial Activity Assays

The ADD in vitro method was performed to evaluate the antibacterial activity of EO and BAK solutions. An amount of 10 µL of each bacterial broth culture (normalized to the concentration of 1 × 106 CFU/mL) was uniformly spread using a sterile Drigalsky spatula on the nutrient agar surface, and the surface was allowed to dry (1 h at 30 °C). A sterile paper disc (6 mm in diameter, Dutscher papier, FR) imbibed with 10 µL of the *T. vulgaris* essential oil (100% or 50%) or control solutions (BAK 3% *v*/*v* and pentane 100%,) was leaned on the agar medium (9 cm Petri dishes) surface. Due to the non-miscibility of essential oils in water, fifty percent of the EO solution was composed of pentane. After incubation at 30 °C for 24 h, inhibition halos (i.hs.) differing in diameter (mm) were revealed, reflecting the antimicrobial activity and categorized as a sensitive strain (i.h. > 9 mm) or a resistant strain (i.h. < 9 mm). *Georgenia* sp. colonies were the most susceptible isolated bacteria, showing a relevant sensitivity to the essential oil with an inhibition zone of up to 18 mm. At the other end of the spectrum, *Bacillus* sp. was the most resistant with an i.h. of 14–17 mm diameters. Table 2 shows the results of the antibacterial activity using the ADD method (Figure 1). Pentane, accounting for fifty percent of the EO solution, was applied as a control to evaluate the real biocide power of the diluted EO, and it did not show inhibition halos.

### 3.6. Vacuum Chamber

In order to maintain stable thermo-hygrometric parameters (13–18 °C; 50–60% RH), a saturated solution of MgCl_2_ was placed inside the vacuum system. In addition, the chamber was filled with the *Thymus vulgaris* EO, calculating the right amount to saturate atmosphere using the equation of a perfect gas; a thermo-hygrometer; and the leather samples, placed on a reticulated support made of cardboard and nylon to allow the volatile compounds to easily reach all points of the samples. To create vacuum, a diaphragm vacuum pump (Type MZ 2C, Ser.No. 20635805) was connected by a tube to the top of the chamber and was kept running for about one minute and then disconnected.

## 4. Conclusions

The present work focused on determining the yield, the chemical composition and antimicrobial properties of *Thymus vulgaris* and *Crithmum maritimum* essential oils. The essential oil of *T. vulgaris* was characterized by a large presence of monoterpenes, *p*-cymene (35.96%), terpinen-4-ol (10.29%), *α*-terpinene (8.85%) and thymol (25.38%), while *C. maritimum* essential oil was dominated by *β*-myrcene (13.66%), followed by *p*-cymene (11.67%), *β*-phellandrene (6.57%) and thymol acetate (14.38%). In addition, we suggested an integrated approach to the most common disinfection processes, by using a vacuum chamber in order to allow the mechanism to act faster than usual. *Thymus* oil, in vapor phase, is a strong inhibitor for bacterial growth; every bacterial colony isolated showed significant sensitivity to both EO solutions (50%, 100%) and was able to produce inhibition halos up to 33 mm. All colonies under vacuum conditions were significantly reduced compared to the same ones exposed to environmental conditions. In contrast, the *C. maritimum* essential oil did not show inhibition halos. The potential use of commercial plant essential oils, together with the achievements reached during the in vitro and in situ applications to control the growth of bacterial taxa, led us to hypothesize their use as natural biocides, replacing the most common toxic biocide usually used in the conservation of cultural heritage.

## Figures and Tables

**Table 3:** Antimicrobial activity of *T. vulgaris* EO using ADD method.

EO/Solvents	Conc. (%)	Inhibition Halos (mm) ^a^ × Bacterial Specie			
		*Bacillus*	*Georgenia*	*Streptomyces*	*Ornithinibacillus*
*T. vulgaris* EO	50	33	confl. ^b^	42	39
	100	38	confl. ^b^	46	54
*C. maritimum* EO	50	9	0	0	0
	100	10	0	0	0
Benzalkonium chloride	3	7	9	8	6
Pentane	100	0	0	0	0

^a^ Inhibition halo diameter, including paper disk diameter (6 mm). Sensitive strains > 9 mm; not sensitive < 9 mm. ^b^ Confluent: microbial growth on the entire surface of agar medium.
